# The effect of perceived responsibility on stigma toward people experiencing food insecurity and accessing food support: an experimental vignette study

**DOI:** 10.3389/fpubh.2026.1750930

**Published:** 2026-04-08

**Authors:** Natalie Taylor, Jenna R. Cummings, Paul Christiansen, Emma Boyland, Charlotte A. Hardman

**Affiliations:** Department of Psychology, University of Liverpool, Liverpool, United Kingdom

**Keywords:** attribution theory, familiarity, food bank, food insecurity, stigma, vignette

## Abstract

**Introduction:**

University students are disproportionately at risk of food insecurity, and subsequent poor physical and mental health and low academic performance. However, students may not access food support due to associated stigma. Stigma toward people experiencing food insecurity is thought to partly originate from negative media stories which highlight the driving role of internal responsibility, despite research suggesting that external factors are more culpable. Grounded in attribution theory, this pre-registered (https://doi.org/10.17605/OSF.IO/9X7M2) online experiment aimed to examine the effect of perceived responsibility for food insecurity and food support access on stigmatizing attitudes, namely cognitive beliefs, affective reactions and discriminatory inclinations.

**Methods:**

Participants (*N* = 322, *N* = 106 self-reporting as food insecure, all university students over 18 years old), were randomly assigned to one of three conditions requiring them to read a bogus newspaper article framing food insecurity and food support access as originating from (i) perceived internal responsibility; (ii) perceived external responsibility; or a (iii) control article with no responsibility framing. Then participants read a standard vignette regarding a fictional character who was experiencing food insecurity and accessing a food bank, before completing measures of stigma toward the fictional vignette character.

**Results:**

There were no statistically significant effects of condition on stigma toward the fictional character.

**Discussion:**

However, methodological issues could have contributed to these null findings, including an insufficient experimental manipulation and use of self-reported measures to quantify stigmatizing attitudes which are vulnerable to social desirability. Future research might explore the research aims using repeated exposures to media stories and behavioral over self-report measures of stigma.

## 1 Introduction

### 1.1 Background

Populations experiencing food insecurity in high income countries are characterized by an “inability to acquire or consume (financially or otherwise) adequate quality or sufficient quantity of food in socially acceptable ways, or an uncertainty that one will be able to do so” (1). Food insecurity is commonly considered to be experienced across a continuum, ranging from low food insecurity, in which food can be accessed with little difficulty, to marginal food insecurity, in which anxiety and worry over food is prevalent without affecting usual eating patterns, to high food insecurity, involving disruptions to usual eating patterns such as skipping meals and going hungry (2, 3). Despite decreasing since January 2025, over 11% of the UK population still reported experiencing food insecurity in June 2025, equivalent to around six million adults (4). As a result, food insecurity remains a significant public health concern, with research showing the significant negative impacts upon not only physical, but also mental health and wellbeing (5–8).

University students are a subgroup of the general population who are specifically vulnerable to food insecurity (9–11). For example, Yau et al. (10) found that full-time university students in the UK were over three times more likely to be experiencing food insecurity than non-students. Higher rates of food insecurity among university students have been attributed to the neoliberalisation of higher education, shifting financial responsibility onto students and increasing financial pressures (12). Coupled with diminished financial support and uncertainty regarding stable and relevant employment prospects upon graduation (12, 13), it is clear why university students are especially at risk. Additionally, attending university is a unique period in which students start to live independently as adults, becoming responsible for their own food and eating decisions (11, 14, 15).

Across other high-income countries, food insecurity within students has been linked to unhealthy eating behaviors including meal skipping and consumption of fast food, takeaways and sugar sweetened beverages, worse physical and mental health outcomes, and lower academic performance than food secure counterparts (9, 11, 16–20). Alongside often experiencing a lack of focus when studying (19, 20), students identifying as experiencing food insecurity may be missing out on contact time at university in order to work and earn more money to alleviate their food insecurity, further affecting their academic achievement and exacerbating stress (14, 21). Additionally, university students with food insecurity have reported feeling stigmatized regarding their food circumstances (14). For example, research has found that university students experiencing food insecurity felt less comfortable talking about their food circumstances to counseling and wellness staff, federal social services, and their parents if they were to experience food shortage, compared to their food secure counterparts (11). This suggests a preference to conceal food-related struggles in university students, a concept considered to be theoretically related to food insecurity stigma (22). Moreover, Harville et al. (11) found that only 13% of university students experiencing food insecurity had accessed a campus-based food bank, with Henry (14) reporting that students emphasized the need for discretion and confidentiality in relation to use of such support services. Taken together this indicates that university students experiencing food insecurity may avoid accessing sources of food support due to the associated shame and stigma from peers (14), which may further impact mental wellbeing, academic performance, and future prospects (23). However, little is known about the underpinning processes of stigmatization (including contributions of different types of stigma, such as public, self-, or anticipated stigma) in the context of food insecurity and food support access within university student populations.

### 1.2 Food insecurity and food support stigma and attribution theory

Attribution theory is an important framework for understanding the pathway between stigmatization and negative or discriminatory behavior (24). The theory suggests that people use information regarding the perceived controllability of a stigmatized condition to assign internal (i.e., responsibility of the individual for their stigmatized condition) or external (i.e., responsibility of external factors out of the control of the individual) responsibility. This information is then used to shape their cognitive beliefs about the condition, defined as beliefs regarding the causes of a person's behavior (such as endorsement of stereotypes, blameworthiness, and deservingness) (25). In turn, this influences their affective reactions, defined as emotional responses to such beliefs (such as fear and anger, or pity and sympathy), giving rise to discriminatory or helping inclinations, defined as behavioral tendencies based on cognitive beliefs and affective reactions which reinforce stereotypes and prejudice (such as likelihood of donating and desire for social distance). The theory has been commonly used across the literature in areas including obesity, mental health and poverty stigma (26–33). For example, in the poverty stigma context, poverty ascribed to laziness and low motivation (i.e., internal responsibility) would elicit more negative affective reactions such as anger and discriminatory inclinations such as unwillingness to help, resulting in a higher level of stigmatization than if poverty was ascribed to external structural factors (i.e., external responsibility), which would lead to more sympathetic responses and willingness to help (24, 32, 33).

However, attribution theory in the context of food insecurity and food support access is underexplored, despite its potential relevance to further understanding stigma. The dichotomy between internal and external responsibility has direct relevance to ongoing discourses within the food insecurity literature, which highlight a divide between those who perceive food insecurity to have internal cause, and thus internal responsibility vs. those who perceive external causes, and thus external responsibility. A recent systematic review suggested that food insecurity is largely driven by external, structural factors including market and government failures, which are out of the control of people experiencing food insecurity (5). However, over the last decade, neoliberal discourses which shift responsibility for the effects of austerity—including food insecurity and food support use—from the state to the individual have initiated and reinforced public stigmatization, driving perceptions of internal responsibility (8, 34–36). For example, these neoliberal discourses have become normalized into pejorative stereotypes of people experiencing food insecurity and accessing food support including laziness, fecklessness, undeservingness, lack of education or concern for health, an inability to implement skills such as budgeting and cooking, and a lack of motivation to improve their situation (34, 37, 38), which are broadly misrepresentative (39–43). These negative beliefs and perceptions may be largely formed from what is reported in the mainstream media, with news media in particular having the potential to shape public opinions and reflect certain political agendas, contributing toward increased stigmatization (40, 44–46). Subsequently, perceptions of internal responsibility for food insecurity have been reported not only by the public, but worryingly, also by staff at referral agencies, and food support service volunteers (36, 46, 47).

Despite increasing recognition of food insecurity and food support stigma and the associated negative psychological and physical health impacts, to our knowledge, no quantitative studies have explored how cognitive beliefs, affective reactions and discriminatory inclinations toward people (and particularly university students) experiencing food insecurity and accessing food support might be influenced by perceived responsibility. Increasing understanding of the drivers of stigmatization is important and may contribute toward developing new ways to communicate with the public around food insecurity and food support access, as well as advocating for people experiencing acute forms of food insecurity in a way that prioritizes psychological wellbeing.

### 1.3 Familiarity in the context of food insecurity

Familiarity is defined as the knowledge, understanding, or personal or vicarious experience of a condition (48) and may also influence the development of stigmatizing attitudes and discriminatory behavior. In the context of mental health stigma, Corrigan and Nieweglowski (49) found that 73% of studies reported higher familiarity with mental health to be associated with less stigma (i.e., less negative cognitive beliefs, less negative affective reactions and fewer discriminatory inclinations). Research indicates that familiarity may reduce stigma within groups if in-group members do not endorse negative stereotypes about their own group (50). In this sense, people experiencing food insecurity and accessing food support may stigmatize other in-group members less than out-group members might.

However, Corrigan and Nieweglowski (49) found that a small number of studies also showed the opposite association; familiarity with mental illness was associated with producing a higher perceived family burden (defined as the impacts of having a family member with a mental illness, including impacts to finances, health, relationships and quality of life) and initiating associative stigma toward people experiencing mental illness. Evidence suggests that people experiencing food insecurity and accessing food banks create an imaginary undeserving and feckless “Other” who opposes their own deserving and worthy values (51). This may be explained through social identity theory, which suggests that members of lower status social groups identify less with their own social group in order to distinguish themselves and improve their self-concept (50, 52–54). Within people experiencing food insecurity and accessing food support, this may lead to a hierarchy of status within the in-group, resulting in some individuals experiencing food insecurity and accessing food support being seen as more or less worthy than others (39, 41, 51). This may trigger stigmatizing cognitive-emotional processes toward others of the same condition and suggests a potential positive association between familiarity and stigma. Additionally, when exploring the role of familiarity in mental illness stigma, Corrigan et al. (28) found that while familiarity was associated with less negative affective reactions, no association was found between familiarity and attributions of responsibility for the condition. That considered, understanding the influence of familiarity with food insecurity on the association between responsibility attributions and stigmatization should also be prioritized.

### 1.4 Aims and hypothesis

In a sample of university students in the UK, this study applied attribution theory (24) to investigate how manipulation of beliefs about the perceived responsibility for food insecurity and food support access influences stigma (including cognitive beliefs, affective reactions and discriminatory inclinations) applied to a fictional vignette character (an individual named “Alex”) experiencing food insecurity. Beliefs were manipulated using bogus newspaper articles that framed food insecurity and food support access as originating from (i) perceived internal responsibility; (ii) perceived external responsibility; or a (iii) control article with no responsibility framing. It was hypothesized that the manipulation toward perceived internal responsibility for food insecurity would lead to more stigma toward the fictional character compared to the manipulation toward perceived external responsibility and the control condition. It was also hypothesized that the manipulation toward perceived external responsibility for food insecurity would result in less stigma toward the fictional character compared to the control.

Additionally, exploratory analyses aimed to understand the role of participants' own food security status (familiarity) on stigma toward the fictional character. It was hypothesized that participants who self-identified as experiencing food insecurity would show less stigma toward the fictional character compared to participants self-identifying as food secure, replicating the direction observed in Corrigan and Nieweglowski's (49) review of mental illness and stigmatization. Finally, exploratory analysis also aimed to investigate whether familiarity moderated any effects of perceived responsibility for food insecurity and food support access on stigma.

## 2 Materials and methods

### 2.1 Participants and recruitment

The study was advertised as a “Perceptions of employability among students during the cost-of-living crisis” study on posters across a university campus in the Northwest of England, on social media and via word of mouth. Employability was used as a cover story (31) to disguise the true purpose of the study. Participants were eligible to take part in the study if they were students currently studying at a UK university. There were no other eligibility criteria due to the age and English language ability stipulations of entry into UK universities. Due to a lack of previous research in this area, we based the effect size on the effect size found by Ruddock et al. (31) in their paper using similar vignette methodology to apply attribution theory to the context of weight-related stigma and food addiction (ηp^2^ = 0.040; small-medium effect size). Subsequently, GPower version 3.1 (81) was used to conduct a power calculation for a one-way ANOVA using a small-medium effect size (*f* = 0.18) across three groups, giving a total required sample size of *n* = 303 participants (*n* = 101 per condition).

Participants were reimbursed for their time and contribution through voluntary entry into a prize draw to win online shopping (Amazon) vouchers (1st prize £100; 2nd prize £50; 3rd prize £25). Recruitment and data collection took place between July 29th and September 23rd 2024. Ethical approval was granted by the University Central University Research Ethics Committee (reference: 13977) prior to conducting the study. The study was pre-registered on the Open Science Framework before data collection commenced (https://doi.org/10.17605/OSF.IO/9X7M2).

### 2.2 Measures

#### 2.2.1 Attribution measures

**Figure 1:**
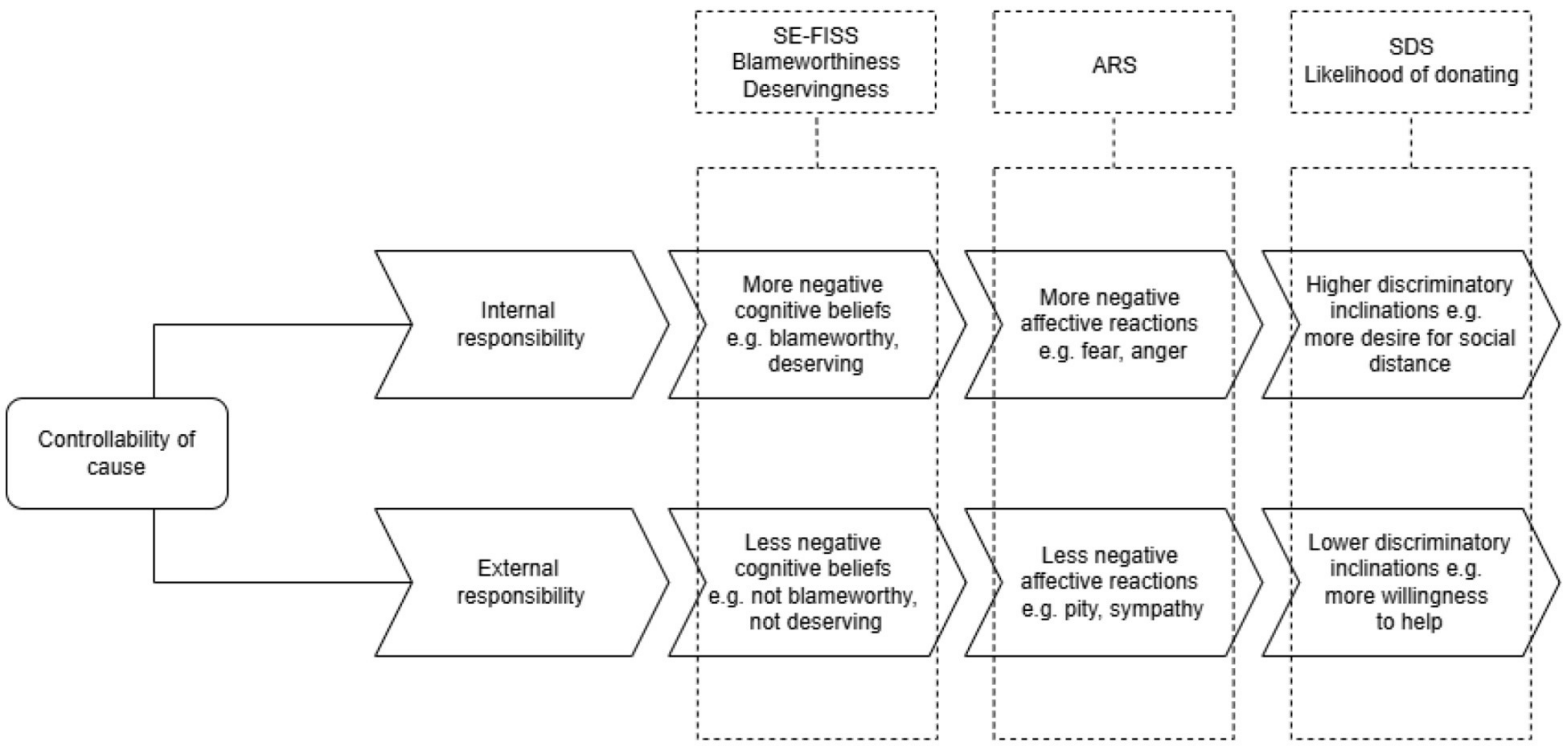
Visual representation of attribution theory [devised from Weiner (24)], showing the six attribution measures mapped onto the relevant underlying construct. SE-FISS, stereotype endorsement subscale of the food insecurity self-stigma scale; ARS, affective reaction scale; SDS, social distance scale.

Six measures spread across three concepts (cognitive beliefs—three measures; affective reactions—one measure; discriminatory inclinations—two measures) were used to indirectly measure stigma (24) (see Figure 1).

##### 2.2.1.1 Cognitive beliefs

###### Stereotype endorsement

The “Stereotype endorsement” subscale of the Food Insecurity Self-stigma Scale (SE-FISS) (22) was used to assess the extent to which participants internalized negative stereotypes about people experiencing food insecurity. Participants were asked to rate (across a five-point Likert scale from “Strongly disagree” to “Strongly agree”) how much they agreed with three statements each focusing on a common stereotype applied to people experiencing food insecurity [(i) “People who have difficulty accessing enough food should take on extra work to improve their situation”; (ii) “People who have difficulty accessing enough food should try to improve their cooking skills to improve their situation”; (iii) “People who have difficulty accessing enough food should try to improve their budgeting skills to improve their situation”]. A total score was calculated (between three and 15), with higher scores indicating greater endorsement of negative stereotypes. Internal reliability of the measure was assessed using McDonald's omega total (ω_t_), with a score of 0.7 or higher considered acceptable (SE-FISS ω_t_ = 0.75) (55). As the three items within the SE-FISS frame food insecurity as a personal responsibility, with participants required to indicate how much they agree or disagree, the SE-FISS measure was used as a manipulation check in the study to assess whether the experimental newspaper articles had the intended effect of shifting perceptions. For example, it was expected that participants in the perceived internal responsibility condition would score significantly higher on the SE-FISS, indicating greater endorsement of negative stereotypes and more perceived internal responsibility for food insecurity than the perceived external responsibility and control conditions. Similarly, it was expected that participants in the perceived external responsibility condition would score significantly lower than the perceived internal responsibility and control conditions.

###### Blameworthiness

To measure blameworthiness, participants were asked to indicate, across a five-point Likert scale from “Strongly disagree” to “Strongly agree,” to what extent they agreed that the fictional vignette character is “to blame for their food insecurity.” This measure was developed for the present study by adapting Sattler et al.'s (27) measure of blameworthiness used in a similar vignette study measuring stigmatization in the context of the COVID-19 pandemic. A higher score (between one and five) indicated more attribution of blame toward the individual (ω_t_ not computed as this measure only had one item).

###### Deservingness of food insecurity and reliance on food support

A two-item measure of deservingness of food insecurity and reliance on food support (hereon referred to as simply “deservingness” for brevity) was also adapted from Sattler et al. (27). Participants were asked to indicate to what extent they agreed that the fictional vignette character deserves: (i) “to experience food insecurity”; and (ii) “to have to rely on food support.” The measure was again scored across a five-point Likert scale from “Strongly disagree” to “Strongly agree” with higher total scores (from 2 to 10) indicating more deservingness of food insecure status and need to rely on food support attributed to the individual (ω_t_ not computed as the deservingness measure had only two items).

##### 2.2.1.2 Affective reactions

###### Negative affective reactions

To assess participants emotional reactions to the fictional vignette character, the Affective Reactions Scale was used (56), as in research exploring mental health stigma (57). The scale consists of 10 pairs of antonyms (e.g., “supportive” vs. “resentful”) and for each antonym pair, participants are asked to rate how they feel toward the vignette character across a seven-point scale. Five items were reverse scored as standard for the ARS, in order for inter-correlations to remain positive between scale items (56). A higher total score (from 7 to 70) indicates more negative affective reactions toward the vignette character (ARS ω_t_ = 0.91).

##### 2.2.1.3 Discriminatory inclinations

###### Social distance

To measure participants' desire for social distance from the fictional vignette character, the Social Distance Scale (SDS) was used (58), a validated and widely used proxy measure of discrimination within stigma research (48, 56, 57, 59). The scale contains seven items (e.g., “Would you like to move next door to a person like Alex?”) and participants are asked to indicate their response on a five-point scale from “Definitely willing” to “Definitely unwilling.” Mean scores are taken across the seven items. Higher mean scores (from one to five) indicate more desire for social distance from the fictional vignette character (SDS ω_t_ = 0.93).

###### Likelihood of donating

To assess likelihood of donating, a measure was taken from a vignette study by Tremblay-Boire and Prakash (60). Participants were asked “How likely are you to donate to a food bank or food aid charity?”, indicating their response on a five-point Likert scale from “Extremely unlikely” to “Extremely likely” (60). For this measure, a lower score (between one and five) indicated a lower likelihood of donating, thus less willingness to help (ω_t_ not computed as the likelihood of donating measure had only one item).

#### 2.2.2 Familiarity measure

Food security status was used as a proxy for participants' familiarity with the vignette character's food insecure status. It was measured using an adapted version of the United States Department of Agriculture's Food Security Survey Module (USDA FSSM) (22, 61). Participants were asked whether, in the last 6 months, themselves or anyone in their household had: (i) “had smaller meals than usual or skipped meals,” (ii) “ever been hungry but not eaten,” and (iii) “not eaten for a whole day,” because they “could not afford, or get access to food.” Participants responding “Yes” to at least one of these questions were classified as food insecure, while participants responding “No” to all three questions were classified as food secure.

#### 2.2.3 Employability cover story

To maintain the cover story, a measure of employability was included, based on the procedure used by Ruddock et al. (31) in their study on weight-related stigma. Five items were used (e.g., “I think Alex would be motivated to work hard at their job”) and participants were asked to rate responses on a five-point Likert scale from “Strongly Disagree” to “Strongly Agree.” Total scores (between 5 and 25) were calculated. Higher scores indicate stronger perceived employability of Alex (employability ω_t_ = 0.87). The results of the primary and exploratory analyses for the employability measures are provided in Supplementary material 1.

### 2.3 Procedure

The study was conducted online via Qualtrics.com. Participants were required to read an information sheet and provide informed consent by ticking a box on the online survey. Once consent was provided, participants were presented with a bogus newspaper article (see Supplementary material 2). Bogus newspaper articles have been used previously to experimentally manipulate perceived attributions of obesity, another condition highly stigmatized through the media (62–64). Three versions of the bogus newspaper article were adapted from one real article published in UK national newspaper The Independent in December 2023 (65). The newspaper articles differed by the inclusion of a short statement framing the responsibility of food insecurity and food bank access, and quotes from previous qualitative research:

i. In the perceived internal responsibility condition the article attributed increases in food insecurity and food bank access to “low cooking and budgeting skills” as well as “low motivation to improve these skills or find better paid employment to increase their budget,” backed up by two quotes taken from Power et al. (83) and Parr et al. (47).
ii. In the perceived external responsibility condition the article attributed increases in food insecurity and food bank access to “the ongoing cost-of-living crisis” as well as “stagnating wages and insufficient benefits which are failing to keep up with inflation,” with supporting quotes taken from Williams et al. (36) and Lambie-Mumford (84).
iii. In the control condition no responsibility was mentioned, and no quotes were used.

Food banks (synonymous with food pantries in the US) were chosen as the example of a food support service used in the study, being the largest provider of emergency food in the UK (66). One of the three versions of the bogus newspaper article was displayed to each participant using the Qualtrics randomization feature. When the newspaper article was displayed to participants, the button to move onto the next question was hidden for 75 s, preventing participants from continuing with the survey until the time had elapsed. The aim of this was to encourage participants to read the full article before progressing with the survey. This time frame was tested by the lead researcher and was considered long enough to read the bogus article.

After reading the assigned newspaper article, all participants were required to read the same short vignette about a fictional gender-neutral character (“Alex”), who was described as experiencing food insecurity and sometimes accessing a food bank (see Supplementary material 3). Using vignettes is common within attribution theory research to indirectly explore participants' inherent attitudes and is considered an ethical substitute for assessing stigma toward an individual from the real world (27, 67–69). The vignette included a brief description of Alex's hobbies, education and current struggle to access food. The vignette appeared the same for all participants regardless of condition. As with the newspaper article, when the vignette was displayed to participants, the button to move onto the next question was hidden for 15 s, again to encourage participants to read the vignette before progressing with the survey. Fifteen seconds was tested by the lead researcher and was considered sufficient time to read the vignette.

After exposure to the vignette, participants were required to complete the survey measures, which assessed cognitive beliefs, affective reactions and discriminatory inclinations toward the vignette character (24). Participants completed the measures in the following order: SE-FISS (also used as a manipulation check to assess whether the experimental manipulation within the newspaper articles had the intended effect), employability questions, ARS, SDS, blameworthiness measure, measure of deservingness of food insecurity and reliance on food support, and likelihood of donating measure (see Figure 1).

Participants then completed demographic measures including gender identity and ethnicity [self-reported measures using categories devised within Advance HE guidance (70)], age, objective and subjective socio-economic status, year of university study, employment status and living situation. Objective socio-economic status was recorded through a proxy measure of mother's and father's educational level (if known), which has been used previously in a study with students experiencing food insecurity (71). Subjective socio-economic status was recorded through an adapted version of the MacArthur Scale of Subjective Social Status (72), which asked participants to rank their family on a 10-rung ladder which represents where people stand in society according to education, money and employment. After completing the measures, a debrief sheet was displayed which explained the true aim of the study, and participants were offered voluntary entry into the prize draw.

### 2.4 Data analysis

Analysis was conducted in RStudio (version 2023.06.0) (82). The R script and corresponding data files used in the analysis are available on Open Science Framework Storage (https://osf.io/csa42/).

#### 2.4.1 Descriptive statistics

Chi-squared tests were conducted to identify any differences between conditions and food security statuses in gender, ethnicity, objective socio-economic status, employment status, living location, and living situation (categorical variables). Chi squared tests were also conducted to identify differences between conditions in food security status (categorical variable). Kruskal–Wallis tests were conducted to identify any differences between conditions and food security statuses in age (measured in categories), subjective socio-economic status, year of university degree, and time of completion (ordinal variables). Identifying differences between conditions and food security statuses across the demographic measures was vital during randomization to ensure any differences found were due to the independent variables.

#### 2.4.2 Primary and exploratory analyses

Due to independence of groups within the independent variables [condition (three groups) and food security status (two groups)], six multifactorial (3 × 2 between-subjects) ANOVAs were conducted to identify main effects of condition on attribution measures (primary analysis), main effects of food security status on attribution measures (exploratory analysis) and interaction effects of condition and food security status on attribution measures (exploratory analysis). Condition (three levels; perceived internal responsibility, perceived external responsibility, control) and participant food security status (two levels; food insecure, food secure) were the independent variables. Each of the six attribution measures was the dependent variable in separate analyses as follows: (i) stereotype endorsement; (ii) blameworthiness; (iii) deservingness; (iv) negative affective reactions; (v) desire for social distance; and (vi) likelihood of donating. In cases where significant effects were found, pairwise comparisons (Estimated Marginal Means; EMM) were conducted to identify where effects lay. MANOVA were avoided due to lack of applicability with the current data and fundamental problems with the test, such as an inability to protect against Type I error and the creation of a composite dependent variable for analysis optimized based on chance and unique to the sample, impacting on generalisability and interpretation of results, for example through the computation of inaccurate effect sizes (73–75).

## 3 Results

### 3.1 Descriptive statistics

A total of 322 participants completed the study (see Supplementary material 4 for the full sample characteristics). Across the whole sample, 33.9% of participants identified as experiencing food insecurity, almost three times the most recent estimation of food insecurity for UK adults by the Food Foundation (4). Additionally, 61.1% identified as women, 50.3% were aged between 18 and 22, 47.8% identified as being of White British (including English, Scottish, Welsh or Northern Irish) ethnicity. In terms of socio-economic status, 43.1% reported both parents being educated further than high school on the objective measure, while the mean score on the subjective measure (MacArthur Scale of Subjective Social Status (72), representing a 10-rung ladder of social standing based on education, money and employment) was 6 ± 1.87, indicating that participants were objectively, but also subjectively perceived themselves to be, fairly affluent. Participant numbers were evenly spread across conditions (internal: *N* = 108; external: *N* = 106; control: *N* = 108) and there were no significant differences in sample characteristics between conditions or between food security statuses (see Supplementary material 4). The average time to complete the survey was 21.5 min, with no significant differences observed between conditions or between food security statuses.

Mean scores for the attribution measures across the whole sample, as well as stratified for food security status and condition are given in Table 1. Additional descriptives of attribution measure scores are provided in Supplementary material 5.

**Table 1:** Mean scores and standard deviation for the attribution measures.

Attribution measures	Range	Mean (SD)					
		Total	Food security status		Condition		
		*N* = 322	Secure (*N* = 216)	Insecure (*N* = 106)	Internal (*N* = 108)	External (*N* = 106)	Control (*N* = 108)
Cognitive beliefs							
Stereotype endorsement	3–15	9.40 (2.96)	9.38 (2.91)	9.45 (3.06)	9.48 (3.05)	9.23 (3.06)	9.49 (2.78)
Blameworthiness	1–5	2.04 (1.07)	2.00 (0.96)	2.13 (1.28)	2.12 (1.14)	2.01 (1.13)	1.99 (0.94)
Deservingness	2–10	3.39 (2.05)	3.19 (1.80)	3.77 (2.45)	3.61 (2.16)	3.22 (1.97)	3.32 (2.03)
Affective reactions							
Negative affective reactions	7–70	29.8 (9.40)	30.1 (9.47)	29.1 (9.27)	29.8 (10.2)	30.4 (9.32)	29.2 (8.64)
Discriminatory inclinations							
Desire for social distance	1–5	2.13 (0.78)	2.13 (0.81)	2.13 (0.78)	2.19 (0.77)	2.08 (0.80)	2.12 (0.83)
Likelihood of donating	1–5	3.71 (1.02)	3.71 (1.03)	3.72 (1.00)	3.74 (1.04)	3.68 (1.01)	3.72 (1.02)

### 3.2 Primary and exploratory analyses

Levene's tests and Non-Constant Variance (NCV) tests showed the assumptions of homogeneity of variance and heteroscedasticity were met for four of the six dependent variables, specifically stereotype endorsement, negative affective reactions, social distance and likelihood of donating. Blameworthiness showed neither homogeneity of variance nor heteroscedasticity. Meanwhile, deservingness showed homogeneity of variance but not heteroscedasticity. Subsequently, white-adjusted ANOVAs were conducted but no differences in significance levels were found (see Supplementary material 6).

#### 3.2.1 Primary analysis: main effect of condition on attribution measures

No significant main effect of condition (perceived internal responsibility, perceived external responsibility, control) was found for any of the six attribution measures (see Table 2).

**Table 2:** Main effects of condition and food security status, and interaction effects of condition and food security status on the six attribution measures.

Attribution theory concepts	Attribution measure	*F*	df (316)	*p*-value	Cohen's *f*^†^
**Cognitive beliefs**	**Stereotype endorsement**				
	Condition	0.27	2	0.762	0.04
	Food security status	0.06	1	0.808	0.01
	Condition^*^food security status	0.48	2	0.618	0.06
	**Blameworthiness**				
	Condition	0.45	2	0.635	0.05
	Food security status	1.12	1	0.291	0.06
	Condition^*^food security status	0.07	2	0.928	0.02
	**Deservingness**				
	Condition	1.07	2	0.344	0.08
	Food security status	5.77	1	**0.017** ^*^	0.14
	Condition^*^food security status	0.86	2	0.425	0.07
**Affective reactions**	**Negative affective reactions**				
	Condition	0.40	2	0.669	0.05
	Food security status	0.88	1	0.350	0.05
	Condition^*^food security status	1.22	2	0.295	0.09
**Discriminatory inclinations**	**Social distance**				
	Condition	0.47	2	0.627	0.05
	Food security status	< 0.01	1	0.987	< 0.01
	Condition^*^food security status	0.71	2	0.494	0.07
	**Likelihood of donating**				
	Condition	0.10	2	0.905	0.03
	Food security status	< 0.01	1	0.966	< 0.01
	Condition^*^food security status	0.15	2	0.865	0.03

^†^Cohen's f interpretation: small effect size f = 0.10; medium effect size f = 0.25; large effect size f = 0.40 (80). The bold values indicates ^*^p < 0.05.

#### 3.2.2 Exploratory analysis: main effects of food security status on attribution measures

The only significant effect found was for the deservingness measure (see Table 2). *Post-hoc* tests (EMM with a Holm adjustment) found that participants who identified as experiencing food insecurity scored significantly higher on the deservingness measure (*p* = 0.016, *d* = 0.29), indicating that they believed the fictional character to be more deserving of their food insecurity and reliance on food support compared to those who identified as being food secure.

#### 3.2.3 Exploratory analysis: interaction effects of condition and food insecurity on attribution measures

No significant interaction effects were found for any of the six attribution measures (see Table 2).

To explore the level of confidence in the non-significant results, Bayesian analyses were conducted, showing strong evidence for the null hypothesis for all non-significant associations (BF_10_ < 0.333; see Supplementary material 7). In the case of the significant finding for the deservingness measure, Bayesian analysis indicated that the data favored neither the null nor the alternative hypothesis (BF_10_ = 1), suggesting that the significant finding is uninformative (see Supplementary material 7).

## 4 Discussion

In a UK student sample, this study examined the effect of perceived responsibility for food insecurity and food support access on cognitive beliefs, affective reactions and discriminatory inclinations toward a fictional vignette character. Based on attribution theory, in which perceived internal responsibility for a stigmatizing condition is thought to increase stigma toward an individual with the condition (24), it was hypothesized that a manipulation toward perceived internal responsibility would lead to more stigma toward the fictional vignette character compared to both a manipulation toward perceived external responsibility and to the control condition (and similarly, less stigma with a manipulation toward perceived external responsibility relative to the control). However, contrary to this, no statistically significant main effects of condition on stigma toward the fictional character were found. Meanwhile, a significant main effect of food security status was only found for one of the six dependent variables (deservingness), indicating that within the sample, people who were experiencing food insecurity thought that others in food insecurity (i.e., other in-group members) were more deserving of their food insecure condition, a more negative cognitive belief which leads to more stigmatizing attitudes according to attribution theory. This finding is inconsistent with research suggesting in-group members show less stigma toward others who share membership to the group (48, 49) but does align with predictions from social identity theory: members of lower status social groups identify less with their own group to create social distance and improve their self-concept, triggering stigmatizing cognitive-emotional processes toward others with the same condition (50, 52). A similar association has also been found within the poverty stigma literature, with people on low income distinguishing between deservingness and undeservingness to create social distance (53, 54). However, caution and further exploration are required when considering the association found between food security status and deservingness, especially given that assumptions for heteroscedascity were not met, and Bayesian analysis revealed an uninformative association (see Supplementary materials 6, 7). In addition, no significant interaction effects of food security status and the experimental manipulation on stigma toward the fictional character were found, suggesting that any potential effects of the experimental manipulation were not moderated by the participants' own food insecure status.

The design of the present study was based on previous vignette-based research and research using bogus newspaper articles to manipulate attitudes and beliefs about food addiction, in which the manipulation was sufficient to induce significant differences between treatment and control groups (31, 62). However, there are some methodological issues with the current study that could have contributed to the failure to observe significant differences when this approach was applied to the context of food insecurity. Firstly, with regards to the experimental manipulation (i.e., reading of an adapted newspaper article framed to portray internal responsibility or external responsibility for food insecurity, or a control article), scores on the SE-FISS (used as the manipulation check in the study) were not significantly different between conditions, indicating that a stronger manipulation was required. Additional exploratory analysis (unplanned and not within the pre-registration) was conducted on each of the three items on the SE-FISS separately to explore individual main effects of condition and food security status, and their interaction on each item, but no significant differences were found (see Supplementary material 8). While the use of bogus news articles to experimentally manipulate attributions of stigmatized conditions has been effective in previous research (62–64), effects may be subtle (76), and reading one newspaper article which differentially attributes responsibility for food insecurity may not be sufficient to affect stigmatizing attitudes toward individuals experiencing food insecurity and accessing food support. Therefore, future studies might aim to utilize a longer test period involving multiple exposures to reinforce the manipulation, and also conduct a pilot study to confirm the manipulation is successful. As an example, ecological momentary analysis could be used to facilitate repeat exposures to the manipulation over a number of weeks, in real time and via smartphone technology to more closely mimic real-life exposures seen in the media. Additionally, more complex study designs which could integrate different types of manipulations (e.g., short articles, infographics, blogs, videos) across different media streams through social media may further enhance both the strength of the manipulation, the external validity of the study, and may also be effective in ensuring sufficient exposure to the manipulation. In the present study, exposure to the manipulation (e.g., reading of the newspaper articles and vignette) was encouraged through a time requirement set on each element during the online survey. However, it may be possible that despite using the time requirement, participants did not read the articles thoroughly (and were therefore not sufficiently exposed to the manipulation) or randomly clicked through multiple-choice responses to finish the survey quickly (meaning that their inherent attitudes were not considered during survey completion). The shortest completion time for the survey was 4 min, which may not be sufficient to meaningfully absorb the information provided in the newspaper article and vignette and complete all measures thoughtfully. Sensitivity analysis was conducted which excluded responses with extreme completion times, but this did not affect the findings (see Supplementary material 9).

Additionally, limitations in the operationalisation of study measures might have impacted on the (null) findings. Firstly, as a measure of agreement with commonly held stereotypes which frame food insecurity as a personal responsibility, the “Stereotype endorsement” subscale of the Food Insecurity Self-stigma Scale (SE-FISS) (22) was used as a proxy for responsibility during the manipulation check to minimize participant burden. However, as the construct stereotype endorsement may be conflated by moral, emotional and cultural character judgements (77, 78), direct measures of attributions of responsibility (e.g., “To what extent do you think people experiencing food insecurity are responsible for their situation”) and control (e.g., “To what extent do you think people experiencing food insecurity have control over their situation”) may have been more appropriate for the manipulation check and better able to detect subtle cognitive shifts. Furthermore, the SE-FISS was designed for use in populations experiencing food insecurity. Yet, within the current study, only 33.9% of participants actually identified as experiencing food insecurity, further suggesting the need for an alternative manipulation check. However, using measures of specific causal beliefs such as responsibility and control may have jeopardized the cover story and threatened to inflict social desirability bias. Repeating the study using alternative measures as the manipulation check would be useful in gaining further understanding regarding the effectiveness of the manipulation. Secondly, the self-reported measures of cognitive beliefs, affective reactions and discriminatory inclinations operationalised to quantify stigmatizing attitudes in the present study may have been vulnerable to social desirability. Despite the anonymous online nature of the study and the implementation of a cover story, it is possible that the true aims of the study were identifiable by participants. Consequently, participants may have purposefully indicated less stigmatizing attitudes and/or not indicated their true food security status in order for their responses to appear socially desirable. Additional descriptives provided in Supplementary material 5 offer further insights into how social desirability might have influenced the attribution measures. For example, histograms of attribution measure scores indicated that scores on blameworthiness, deservingness, and desire for social distance measures clustered toward the lower ends of the scale across conditions and food security statuses, while scores on the likelihood of donating measure skewed toward the top end. The clustering patterns suggest that social desirability may have introduced floor and ceiling effects, with responses aligning with social expectations despite the manipulation. Finding novel, yet ethical ways to explore stigma, including measures of actual observed behavior, rather than self-reported attitudes may be a key focus for future research. One suggestion from the literature for exploring stigma indirectly is using error-choice methods. Within error-choice methods, each fact-based multiple choice question offers erroneous response options which lean toward certain attitudes regarding the stigmatized condition and participants responses are analyzed to elucidate their implicit biases and prejudices (76, 79). Through this indirect approach to measuring stigma, error-choice methods are more capable of bypassing influences of social desirability, as participants are not aware that their implicit prejudices are being tested, as opposed to their knowledge (79). Corrigan et al. (76) used manipulated newspaper articles differing in their framing of mental illness (positive, negative and neutral) to explore stigma toward people experiencing mental illness. The positively framed article significantly reduced stigma, while the negatively framed article significantly increased stigma (76). However, the authors did not find significant effects when using traditional self-report measures of stigma, such as blameworthiness (as in the present study), but only when using the Stigma Through Knowledge Test (STKT), an error-choice method. Future research may aim to apply error-choice methods to the current study design in replacement of the more traditional attribution measures to better understand the role of perceived responsibility in the manifestation of stigmatizing attitudes and discriminatory behavior toward people experiencing food insecurity and accessing food support.

Considering the limitations to the study design highlighted above, the association between perceived responsibility and stigma toward people experiencing food insecurity and relying on food support requires further exploration. There is a lack of quantitative research exploring stigma in this context, particularly in the university student population, and as such, the present study represents preliminary findings in this area. Future research might aim to use the design of the present study as a blueprint, making improvements such as a stronger manipulation, and use of error-choice measures of stigma with attention checks to further test out the study hypotheses. Additionally, cluster analysis could also be applied to reveal sub-group effects. Finally, personal experience of food insecurity was used as a measure of familiarity with food insecurity. Future research might use a measure that also elucidates familiarity through vicarious experiences (e.g., knowledge of friends or family members' experiences with food insecurity and accessing food support). Strengths of the study include the novelty of the application of vignette methodology and attribution theory to the research context and the thoroughness of the method used, including randomization of participants to each condition, quality control measures to encourage reading of the newspaper article and vignette, adequate recruitment and power, pre-registered analyses, and the use of validated measures which are consistent with previous research applying attribution theory to explore stigma. Additionally, the use of sensitivity analysis and Bayesian analyses promoted rigor during analysis.

## 5 Conclusion

The study attempted to experimentally manipulate beliefs about perceived responsibility for food insecurity and access to food support; however, there were no meaningful significant effects of this manipulation on stigma toward the fictional character. The study makes an important first step in the development of quantitative methods to understand potential determinants of stigma toward those facing food insecurity. However, the study design was likely limited by methodological issues including an insufficient experimental manipulation and use of self-reported measures to quantify stigmatizing attitudes. Developing and applying ethical and appropriate research methodology in this field may promote further understanding of how best to address negative stereotypes of food insecurity and food support access within the public domain. Exploring alternative ways to alter beliefs about responsibility for food insecurity (e.g., via repeated exposures to media stories) and using alternative measures of stigma will be important next steps for building on the design of the present study.

## Data Availability

The datasets presented in this study can be found in online repositories. The names of the repository/repositories and accession number(s) can be found below: Open Science Framework, https://osf.io/csa42/.
