# Supplementary material for: The effect of perceived responsibility on stigma toward people experiencing food insecurity and accessing food support: an experimental vignette study
Source: Front Public Health. 2026 Apr 8;14:1750930. doi: 10.3389/fpubh.2026.1750930 (PMC13099287; doi:10.3389/fpubh.2026.1750930)
Supplement: Supplementary file 1 [file Data_Sheet_1.docx]

1. **Results of primary and exploratory analyses for the employability measure**
   1. Descriptive statistics

On average, participants scored slightly higher than the midpoint of 15 on employability (mean score 18.5(3.07)), indicating higher perceived employability of ‘Alex’ (Table SM1.1).

*Table SM1.1. Mean scores and standard deviation of the employability measure. SD: Standard deviation.*

|  | Range | Mean(SD) | | | | | |
| --- | --- | --- | --- | --- | --- | --- | --- |
|  |  | Total | Food security status | | Condition | | |
|  |  | N=322 | Secure  (N=216) | Insecure  (N=106) | Internal  (N=108) | External  (N=106) | Control  (N=108) |
| Employability | 5-25 | 18.5(3.07) | 18.3(2.98) | 18.9(3.23) | 18.2(3.31) | 18.6(2.97) | 18.6(2.92) |

- 1. Primary and exploratory analysis

A multifactorial ANOVA was conducted to identify main and interaction effects of condition and food insecurity on employability. A Levene’s test and NCV test were conducted to test the assumptions of homogeneity of variance and heteroscedasticity respectively. Both assumptions were met for the employability measure.

1.2.1 Primary analysis: Main effects of condition on employability

No significant associations were found between condition (internal responsibility, external responsibility, and control) and employability scores (see Table SM1.2).

1.2.2. Exploratory analysis: Main effects of food security status on employability

No significant associations were found between food security status (food secure or food insecure) and employability scores (see Table SM1.2).

1.2.3. Exploratory analysis: Interaction effects of condition and food security status on employability

No significant interaction effects were found between condition and food security status on employability.

*Table SM1.2. Main and interaction effects of condition and food security status on employability*

| Attribution measure | F | df(316) | p | f |
| --- | --- | --- | --- | --- |
| Employability | | | | |
| Condition | 0.67 | 2 | 0.513 | 0.07 |
| Food security status | 2.30 | 1 | 0.131 | 0.09 |
| Condition*Food security status | 0.97 | 2 | 0.379 | 0.08 |

1. **Experimental materials: bogus newspaper articles**


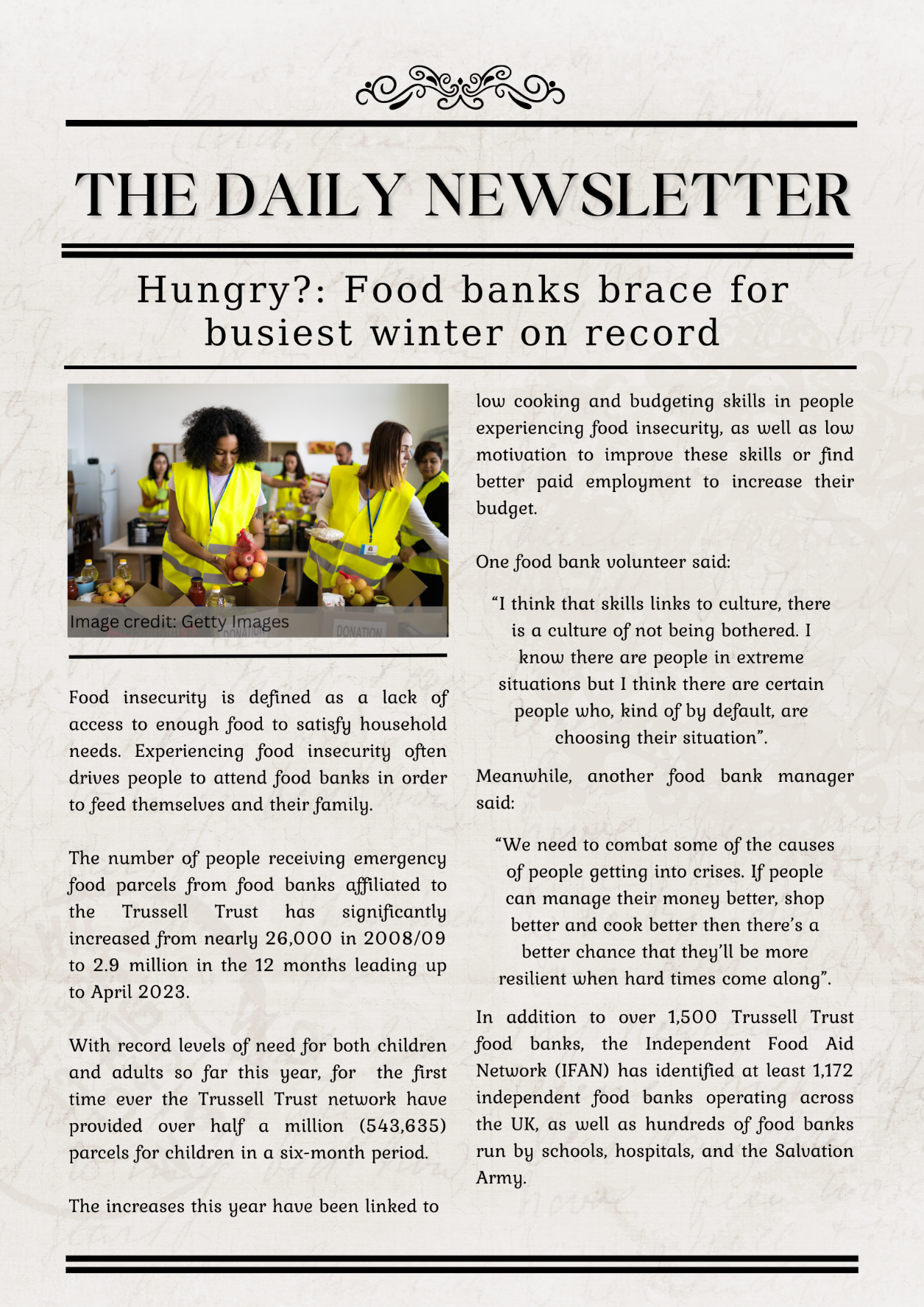
*Article 1: internal responsibility*

*Article 2: external responsibility*


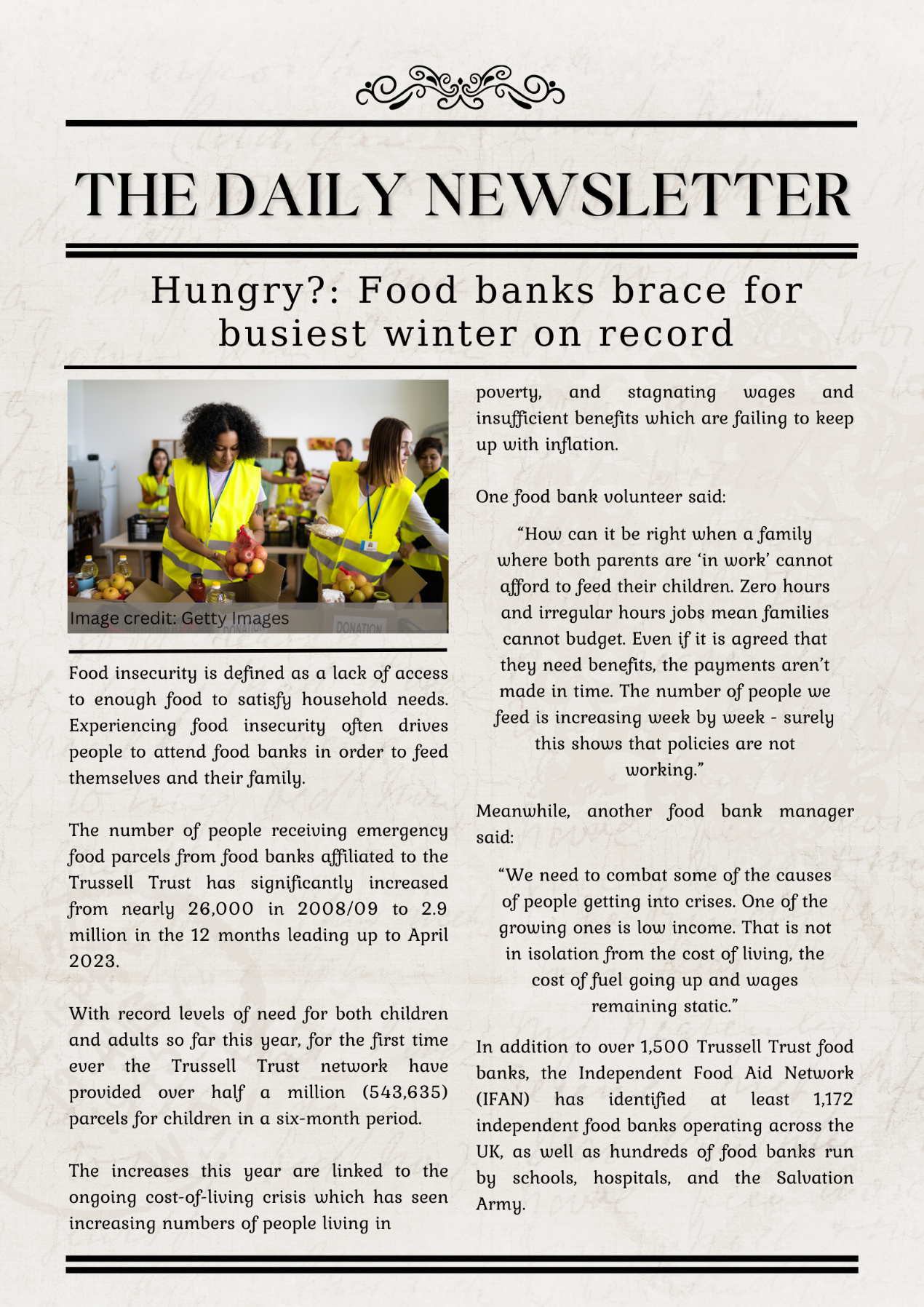


*Article 3: control (no responsibility)*


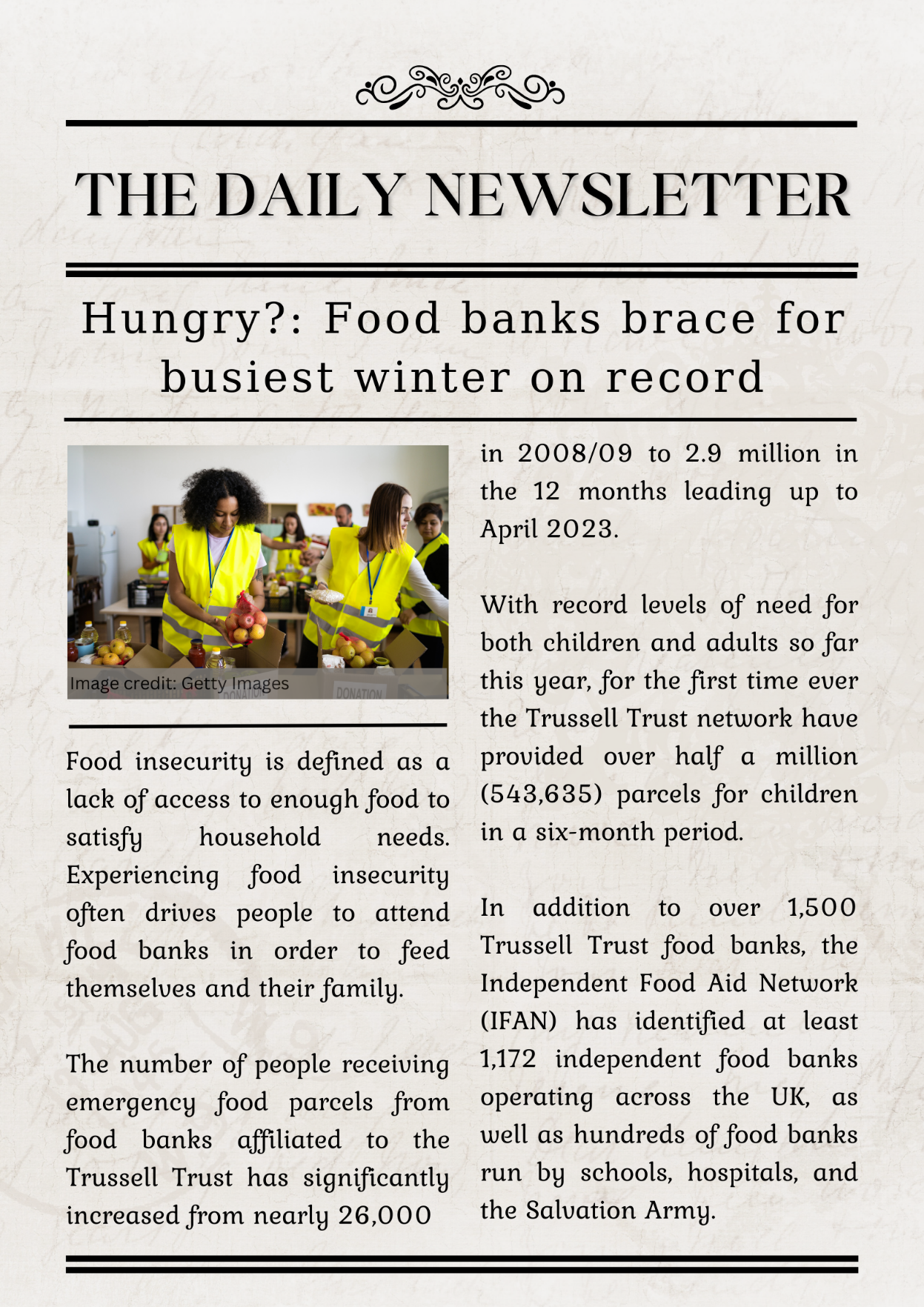


1. **Experimental materials: vignette**

*“Alex is currently in the second year of a university degree in business administration and is aged in the early twenties. Alex moved away from the family home to attend university. Alex has been struggling to afford enough food and has used a local food bank a couple of times. Alex likes running, watching psychological thriller films, and writes short stories as a hobby.”*

1. **Sample descriptive statistics**

*Table SM4.1. Descriptive statistics of the sample*

|  | |  | Total | Food security status | | Condition | | |
| --- | --- | --- | --- | --- | --- | --- | --- | --- |
|  |  |  | N=322 | Secure  (N=216) | Insecure  (N=106) | Internal  (N=108) | External  (N=106) | Control  (N=108) |
| Gender identity | | | | | |  |  |  |
|  | | Man | 90 | 58 | 32 | 30 | 36 | 24 |
|  |  | Woman | 197 | 138 | 59 | 67 | 58 | 72 |
|  |  | Non-binary | 12 | 7 | 5 | 5 | 4 | 3 |
|  |  | Trans | 6 | 2 | 4 | 1 | 2 | 3 |
|  |  | Other | 9 | 8 | 1 | 3 | 3 | 3 |
|  |  | Prefer not to say | 8 | 3 | 5 | 2 | 3 | 3 |
| Age | | | | | |  |  |  |
|  | | 18-22 | 162 | 116 | 46 | 45 | 57 | 60 |
|  |  | 23-27 | 93 | 57 | 36 | 31 | 28 | 34 |
|  |  | 28-31 | 36 | 22 | 14 | 16 | 11 | 9 |
|  |  | 32-36 | 19 | 13 | 6 | 10 | 7 | 2 |
|  |  | 37-41 | 7 | 4 | 3 | 3 | 3 | 1 |
|  |  | 42 and over | 5 | 4 | 1 | 3 | 0 | 2 |
| Ethnicity | | | | | |  |  |  |
|  | | Arab or Arab British | 1 | 0 | 1 | 0 | 1 | 0 |
|  |  | Bangladeshi or Bangladeshi British | 4 | 2 | 2 | 1 | 2 | 1 |
|  |  | Chinese or Chinese British | 20 | 16 | 4 | 7 | 4 | 9 |
|  |  | Indian or Indian British | 9 | 7 | 2 | 2 | 4 | 3 |
|  |  | Pakistani or Pakistani British | 3 | 1 | 2 | 2 | 1 | 0 |
|  |  | African or African British | 4 | 1 | 3 | 1 | 2 | 1 |
|  |  | Caribbean or Caribbean British | 2 | 1 | 1 | 0 | 1 | 1 |
|  |  | White/White British and Asian/Asian British | 43 | 27 | 16 | 16 | 16 | 11 |
|  |  | White/White British and Black African or Black African British | 14 | 7 | 7 | 5 | 3 | 6 |
|  |  | White/White British and Black Caribbean/Black Caribbean British | 4 | 3 | 1 | 2 | 1 | 1 |
|  |  | Other mixed or multiple ethnic background | 12 | 8 | 4 | 2 | 2 | 8 |
|  |  | White English, Scottish, Welsh, Northern Irish or British | 154 | 112 | 42 | 50 | 55 | 49 |
|  |  | White Irish | 5 | 4 | 1 | 2 | 3 | 0 |
|  |  | White Polish | 7 | 1 | 6 | 4 | 1 | 2 |
|  |  | Other White background | 32 | 22 | 10 | 11 | 8 | 13 |
|  |  | Other | 8 | 4 | 4 | 3 | 2 | 3 |
| OSES | | | | | |  |  |  |
|  | | Both parents educated further than high school | 139 | 95 | 44 | 54 | 42 | 43 |
|  |  | One parent educated further than high school and one at high school level or lower | 99 | 63 | 36 | 30 | 39 | 30 |
|  |  | Both parents educated at high school level or lower | 64 | 43 | 21 | 19 | 19 | 26 |
|  |  | One parent educated further than high school and not sure/not applicable/prefer not to say for the other | 7 | 6 | 1 | 1 | 0 | 6 |
|  |  | One parent educated at high school level or lower and not sure/not applicable/prefer not to say for the other | 3 | 3 | 0 | 2 | 1 | 0 |
|  |  | Not sure/not applicable/prefer not to say for both parents | 10 | 6 | 4 | 2 | 5 | 3 |
| SSES | | | | | |  |  |  |
|  | | 1 | 0 | 0 | 0 | 0 | 0 | 0 |
|  |  | 2 | 12 | 7 | 5 | 2 | 7 | 3 |
|  |  | 3 | 25 | 16 | 9 | 10 | 7 | 8 |
|  |  | 4 | 39 | 20 | 19 | 10 | 15 | 14 |
|  |  | 5 | 42 | 24 | 18 | 12 | 10 | 20 |
|  |  | 6 | 58 | 40 | 18 | 26 | 13 | 19 |
|  |  | 7 | 72 | 55 | 18 | 23 | 26 | 23 |
|  |  | 8 | 51 | 40 | 11 | 20 | 17 | 14 |
|  |  | 9 | 20 | 12 | 8 | 5 | 9 | 6 |
|  |  | 10 | 3 | 2 | 1 | 0 | 2 | 1 |
| Year of university degree | | | | | |  |  |  |
|  | | Undergraduate year 1 | 47 | 38 | 9 | 18 | 14 | 15 |
|  | **Living in catered accommodation** | | |  |  |  |  |  |
|  | | Yes | 5 | 3 | 2 | 3 | 2 | 0 |
|  |  | No | 41 | 34 | 7 | 15 | 12 | 14 |
|  |  | Prefer not to say | 1 | 1 | 0 | 0 | 0 | 1 |
|  |  | Undergraduate year 2 | 53 | 30 | 23 | 10 | 21 | 22 |
|  |  | Undergraduate year 3 | 80 | 54 | 26 | 24 | 28 | 28 |
|  |  | Postgraduate masters | 58 | 35 | 23 | 22 | 18 | 18 |
|  |  | Postgraduate PhD | 84 | 59 | 25 | 34 | 25 | 25 |
| Employment status | | | | | |  |  |  |
|  | | Part-time job | 161 | 109 | 52 | 46 | 54 | 61 |
|  |  | Full-time job | 43 | 24 | 19 | 20 | 13 | 10 |
|  |  | No job | 111 | 80 | 31 | 39 | 36 | 36 |
|  |  | Prefer not to say | 7 | 3 | 4 | 3 | 3 | 1 |
| Living location | | | | | |  |  |  |
|  | | On campus | 101 | 71 | 30 | 30 | 30 | 41 |
|  |  | Off campus not with a parent or guardian | 182 | 116 | 66 | 63 | 62 | 57 |
|  |  | Off campus with a parent or guardian | 37 | 27 | 10 | 14 | 14 | 9 |
|  |  | Prefer not to say | 2 | 2 | 0 | 1 | 0 | 1 |
| Living situation | | | | | |  |  |  |
|  | | Living alone | 77 | 50 | 27 | 24 | 22 | 31 |
|  |  | Not living alone | 237 | 162 | 75 | 80 | 82 | 75 |
|  |  | Prefer not to say | 8 | 4 | 4 | 4 | 2 | 2 |
| Food security status | | | | | | | | |
|  | | Food secure | 216 | 216 | 0 | 72 | 69 | 75 |
|  | | Food insecure | 106 | 0 | 106 | 36 | 37 | 33 |

*Chi squared tests were conducted to identify differences in gender, ethnicity, OSES, employment status, living location, living situation and food security status between conditions. No significant differences were found. Chi squared tests were also conducted to identify differences in gender, ethnicity, OSES, employment status, living location, and living situation between food security statuses. No significant differences were found.*

*Kruskal Wallis tests were conducted to identify differences in age, SSES, and year of university degree between conditions and food security statuses. No significant differences were found.*

Table SM5.1. Additional descriptives of attribution measure scores

| Variable | Mean | sd | Median | Min | Max | Range | Skew | Kurtosis | se |
| --- | --- | --- | --- | --- | --- | --- | --- | --- | --- |
| Stereotype endorsement | 9.40 | 2.96 | 10 | 3 | 15.00 | 12.00 | -0.36 | -0.73 | 0.16 |
| Blameworthiness | 2.04 | 1.07 | 2 | 1 | 5.00 | 4.00 | 0.85 | -0.09 | 0.06 |
| Deservingness | 3.39 | 2.05 | 2 | 2 | 10.00 | 8.00 | 1.31 | 0.64 | 0.11 |
| Negative affective reactions | 29.82 | 9.40 | 30 | 10 | 67.00 | 57.00 | 0.27 | 0.39 | 0.52 |
| Desire for social distance | 2.13 | 0.80 | 2 | 1 | 4.43 | 3.43 | 0.42 | -0.42 | 0.04 |
| Likelihood of donating | 3.71 | 1.02 | 4 | 1 | 5.00 | 4.00 | -0.79 | -0.02 | 0.06 |


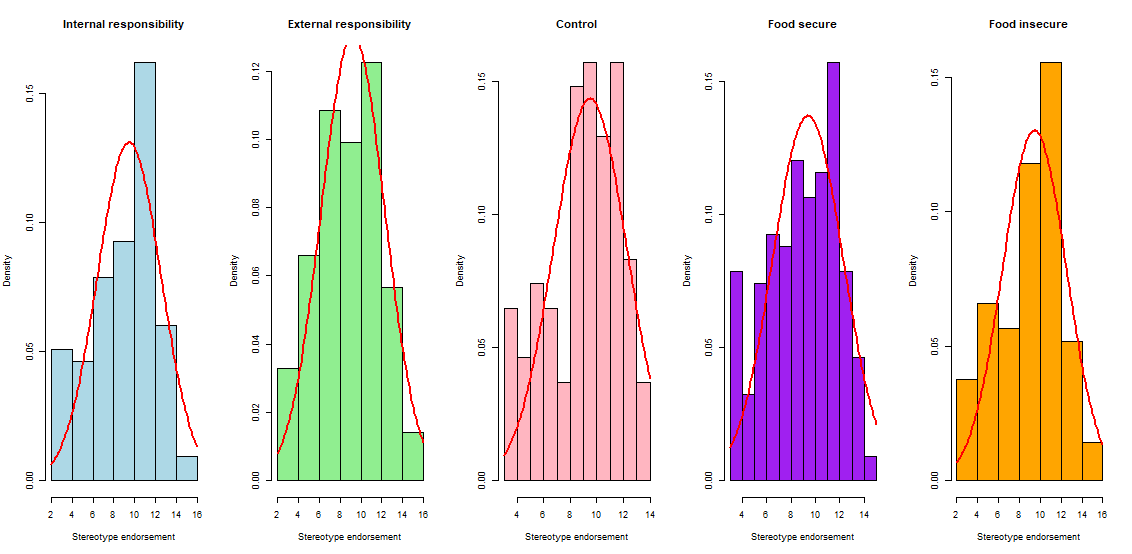


Figure SM5.1. Histogram with normal curve for stereotype endorsement scores by condition and food security status


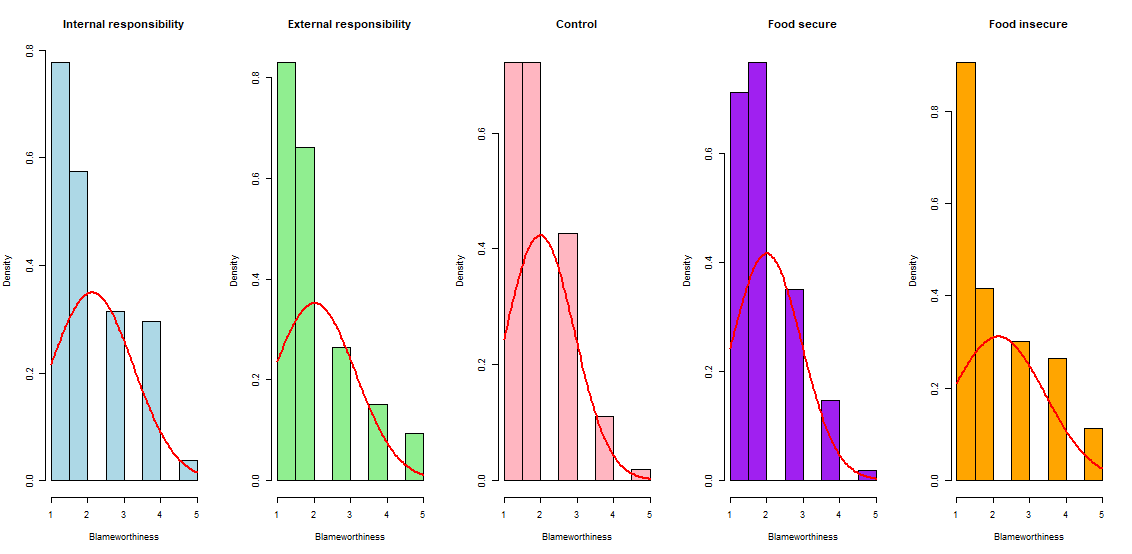


Figure SM5.2. Histogram with normal curve for blameworthiness scores by condition and food security status


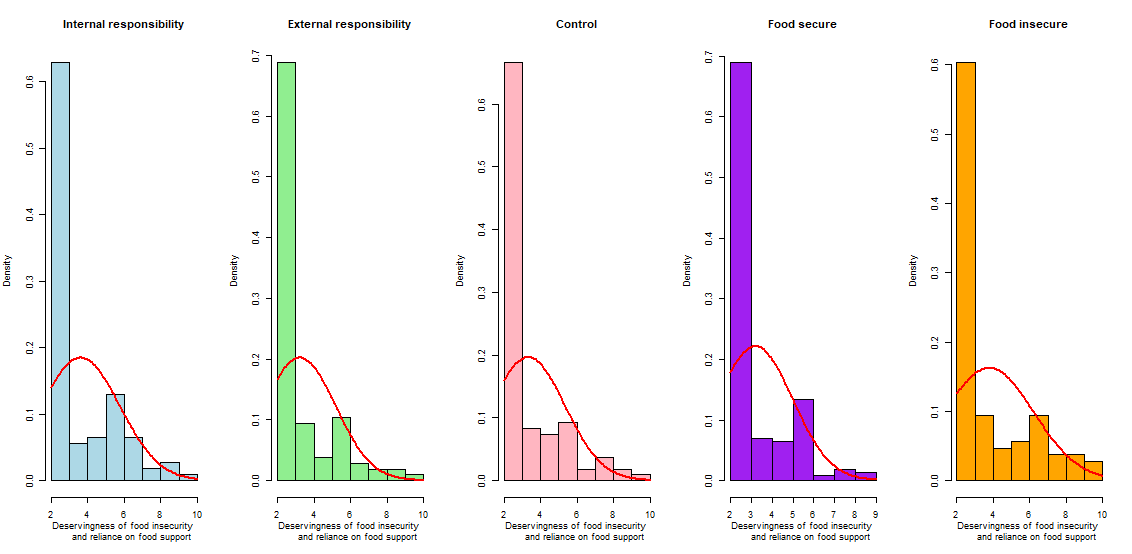


Figure SM5.3. Histogram with normal curve for deservingness of food insecurity and reliance on food support scores by condition and food security status


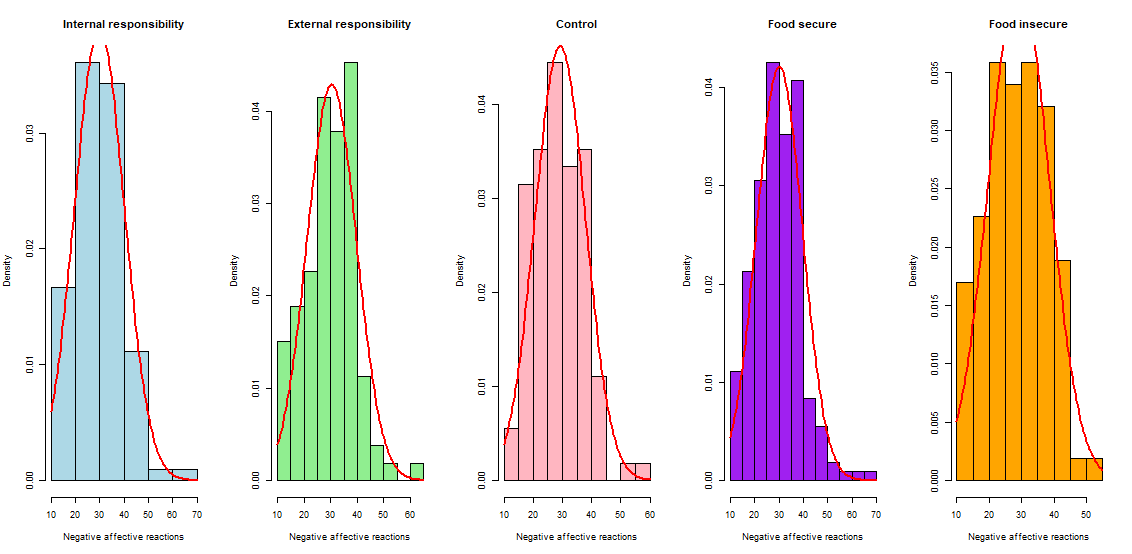


Figure SM5.4. Histogram with normal curve for negative affective reactions scores by condition and food security status


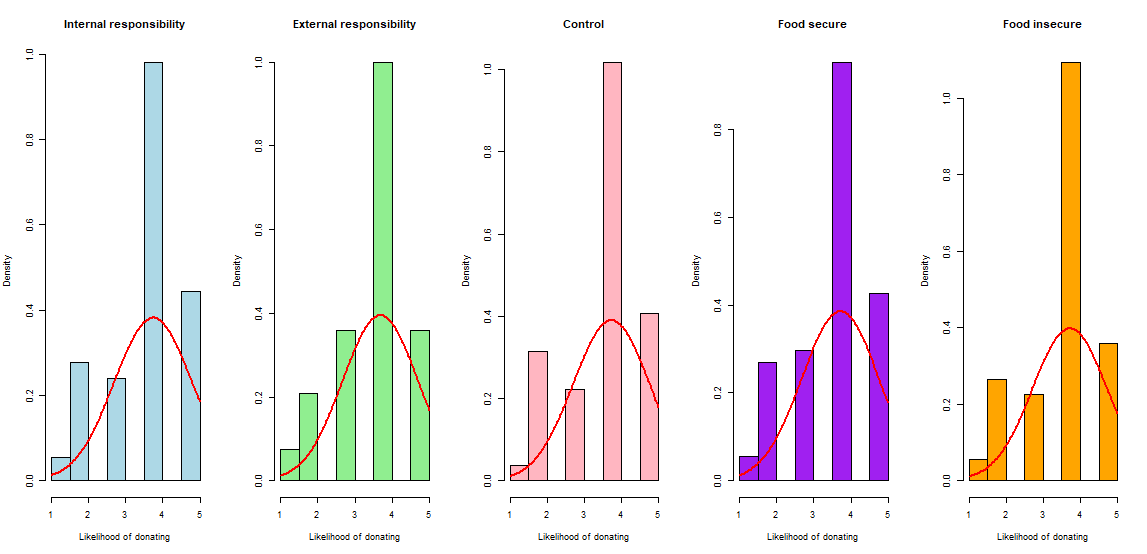


Figure SM5.5. Histogram with normal curve for likelihood of donating scores by condition and food security status


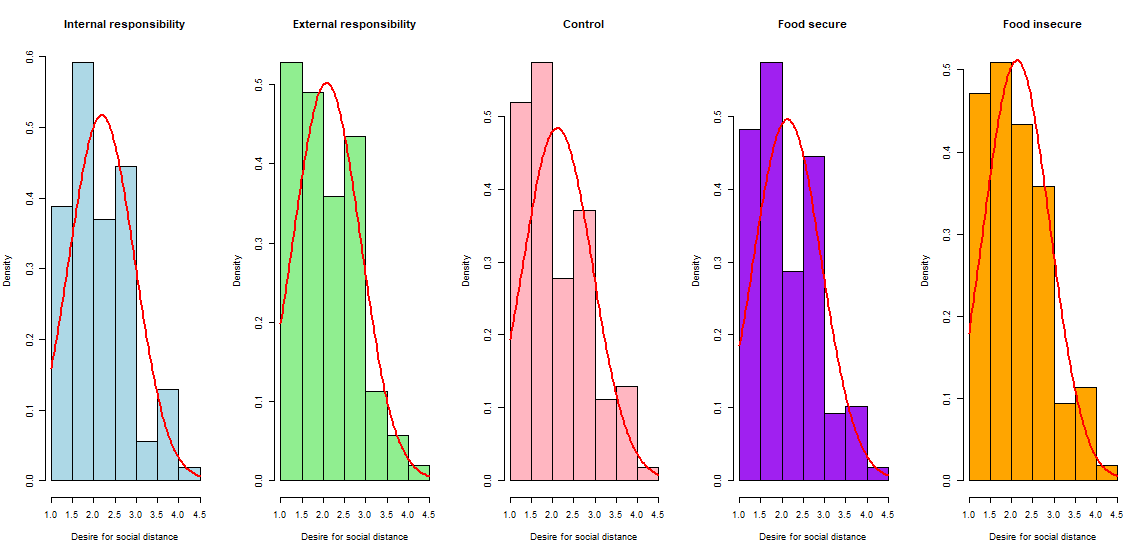


Figure SM5.6. Histogram with normal curve for desire for social distance scores by condition and food security status

**6. Exploratory analysis: White-adjusted ANOVAs**

6.1. Blameworthiness

Assumptions for homogeneity of variance and heteroscedasticity were not met for the blameworthiness measure. Subsequently, a white-adjusted ANOVA was conducted (see Table SM6.1), but still no significant associations were found.

*Table SM6.1. Results of a white-adjusted ANOVA exploring the main and interaction effects of condition and food security on blameworthiness*

| Attribution measure | | F | | df(316) | | p | |
| --- | --- | --- | --- | --- | --- | --- | --- |
| Blameworthiness (white-adjusted) | |  | |  | |  | |
| Condition | 0.56 | | 2 | | 0.573 | |  |
| Food security status | 0.96 | | 1 | | 0.328 | |  |
| Condition*Food security status | 0.06 | | 2 | | 0.942 | |  |

6.2. Deservingness of food insecurity and food support access

Deservingness showed homogeneity of variance but not heteroscedasticity. Subsequently, a white-adjusted ANOVA was conducted (see Table SM6.2). The significant association between deservingness and food security status was still found.

*Table SM6.2. Results of a white-adjusted ANOVA exploring the main and interaction effects of condition and food security on deservingness*

| Attribution measure | | F | | df(316) | | p | |
| --- | --- | --- | --- | --- | --- | --- | --- |
| Deservingness (white-adjusted) | |  | |  | |  | |
| Condition | 0.96 | | 2 | | 0.384 | |  |
| Food security status | 4.17 | | 1 | | **0.042*** | |  |
| Condition*Food security status | 0.70 | | 2 | | 0.497 | |  |

1. **Bayesian analysis**

Bayesian analysis was conducted to gauge the level of confidence that could be drawn in the alternative hypothesis. Suggested conventional cutoffs (Jeffreys, 1998) were used: Strong evidence for the alternative hypothesis given by Bayes factor (BF_10_) > 3; strong evidence for the null hypothesis given by BF_10_  < 0.33̇3; weak or anecdotal evidence given by 0.333 < BF_10_ < 3.

Strong evidence for the null hypothesis was shown for all non-significant associations found during primary and exploratory analysis (BF_10_ < 0.333) (see Table SM7.1).

In the case of the deservingness measure, BF_10_ = 1 indicates that the data neither favours the null or alternative hypothesis, suggesting an uninformative significant result (see Table SM7.1).

In the case of the employability measure, Bayes analysis suggests weak or anecdotal evidence for the null hypothesis, yet this was not further explored, due to employability not being the focus of the current study (see Table SM7.1).

*Table SM7.1. Bayesian analysis of interaction effects ANOVAs*

|  | Attribution measure | BF_10_ |
| --- | --- | --- |
| Cognitive beliefs | **Stereotype endorsement** | |
|  | Condition | 0.043 |
|  | Food security status | 0.133 |
|  | Condition*Food security status | 5.968x10^-4^ |
|  | **Blameworthiness** | |
|  | Condition | 0.051 |
|  | Food security status | 0.226 |
|  | Condition*Food security status | 8.177x10^-4^ |
|  | **Deservingness** | |
|  | Condition | 0.045 |
|  | Food security status | **1.000*** |
|  | Condition*Food security status | 0.012 |
| Affective reactions | **Negative affective reactions** | |
|  | Condition | 0.049 |
|  | Food security status | 0.192 |
|  | Condition*Food security status | 0.002 |
| Discriminatory inclinations | **Social distance** | |
|  | Condition | 0.052 |
|  | Food security status | 0.130 |
|  | Condition*Food security status | 8.432x10^-4^ |
|  | **Likelihood of donating** |  |
|  | Condition | 0.037 |
|  | Food security status | 0.130 |
|  | Condition*Food security status | 3.670x10^-4^ |
|  | **Employability** | |
|  | Condition | 0.062 |
|  | Food security status | **0.386^∆^** |
|  | Condition*Food security status | 0.004 |

**Bayes analysis suggests weak or anecdotal evidence for the nu.*

*^∆^Bayes analysis suggests the effect of food security status favours neither the null or alternative hypothesis.*

1. **Additional exploratory analysis using the SE-FISS**

Table SM8.1. Main effects of condition and food security status, and interaction effects of condition and food security status on individual SE-FISS items

†Cohen’s f interpretation: small effect size f=0.10; medium effect size f=0.25; large effect size f=0.40 (74)

| SE-FISS item | F | df(316) | p | Cohen’s f ^†^ |
| --- | --- | --- | --- | --- |
| Item 1 | | | | |
| Condition | 0.20 | 2 | 0.82 | 0.04 |
| Food security status | 0.02 | 1 | 0.88 | <0.01 |
| Condition*Food security status | 0.51 | 2 | 0.60 | 0.06 |
| Item 2 | | | | |
| Condition | 0.50 | 2 | 0.61 | 0.06 |
| Food security status | 0.30 | 1 | 0.59 | 0.03 |
| Condition*Food security status | 0.42 | 2 | 0.66 | 0.05 |
| Item 3 | | | | |
| Condition | 1.25 | 2 | 0.29 | 0.09 |
| Food security status | 0.02 | 1 | 0.88 | <0.01 |
| Condition*Food security status | 0.21 | 2 | 0.81 | 0.04 |

SE-FISS item 1: “People who have difficulty accessing enough food should take on extra work to improve their situation”
SE-FISS item 2: “People who have difficulty accessing enough food should try to improve their cooking skills to improve their situation”
SE-FISS item 3: “People who have difficulty accessing enough food should try to improve their budgeting skills to improve their situation”

Assumptions for heteroscedasticity and homogeneity of variance were met for each item.

1. **Sensitivity analysis**

Due to some participants registering unexpectedly short or long survey completion times, sensitivity analysis was conducted which excluded responses which took less than 5 minutes and longer than 30 minutes (300-1800 seconds). This time frame was considered appropriate practically (i.e. enough time to feasibly read the newspapers and vignette and respond to the survey measures) and statistically (covering completion times between the 1^st^ and 3^rd^ quartiles and extending beyond the mean (see table SM9.1)).

*Table SM9.1. Spread of completion times for the survey*

|  | Completion time (seconds) |
| --- | --- |
| Minimum | 233 |
| 1^st^ quartile | 417 |
| Mean | 1296 |
| 3^rd^ quartile | 762 |
| Maximum | 71167 |

Conducting this sensitivity analysis did not affect the results (see Table SM9.2).

*Table SM9.2. Main effects of condition and food security status, and interaction effects of condition and food security status on the six attribution measures under sensitivity analysis*

|  | Attribution measure | F | df(316) | p | Cohen’s f |
| --- | --- | --- | --- | --- | --- |
| Cognitive beliefs | **Stereotype endorsement** | | | | |
|  | Condition | 0.32 | 2 | 0.725 | 0.05 |
|  | Food security status | 0.02 | 1 | 0.887 | <0.01 |
|  | Condition*Food security status | 0.42 | 2 | 0.656 | 0.05 |
|  | **Blameworthiness** | | | | |
|  | Condition | 0.33 | 2 | 0.718 | 0.05 |
|  | Food security status | 0.85 | 1 | 0.356 | 0.05 |
|  | Condition*Food security status | 0.04 | 2 | 0.957 | 0.02 |
|  | **Deservingness** | | | | |
|  | Condition | 1.27 | 2 | 0.281 | 0.09 |
|  | Food security status | 4.25 | 1 | **0.040*** | 0.12 |
|  | Condition*Food security status | 0.70 | 2 | 0.500 | 0.07 |
| Affective reactions | **Negative affective reactions** | | | | |
|  | Condition | 0.20 | 2 | 0.815 | 0.04 |
|  | Food security status | 1.15 | 1 | 0.285 | 0.06 |
|  | Condition*Food security status | 0.81 | 2 | 0.445 | 0.08 |
| Discriminatory inclinations | **Social distance** | | | | |
|  | Condition | 0.69 | 2 | 0.503 | 0.07 |
|  | Food security status | 0.22 | 1 | 0.641 | 0.03 |
|  | Condition*Food security status | 0.85 | 2 | 0.429 | 0.08 |
|  | **Likelihood of donating** | | | | |
|  | Condition | 0.11 | 2 | 0.892 | 0.03 |
|  | Food security status | 0.01 | 1 | 0.913 | <0.01 |
|  | Condition*Food security status | 0.14 | 2 | 0.867 | 0.03 |
